# Syncytin-a deficiency compromises murine sperm function by suppressing PRL/PGE2 and PI3K/AKT/mTOR pathway

**DOI:** 10.1016/j.isci.2026.115694

**Published:** 2026-04-12

**Authors:** Qianqian Wang, Zhenwei Wang, Zhenpeng Li, Dongle Liu, Xiaotong Yan, Yan Zhang, Ning Chi, Qiang Bian, Zhankui Zhao, Honglian Yu

**Affiliations:** 1 Department of Pathophysiology, Shandong Second Medical University, Weifang, Shandong 261053, P.R. China; 2 Department of Microbiology and Immunology, Shanghai University of Medicine and Health Sciences, Shanghai 201318, P.R. China; 3 Department of Urology, Seventh People’s Hospital of Shanghai University of TCM, Shanghai 200137, P.R. China; 4 Department of Biochemistry, Jining Medical University, Jining, Shandong 272067, P.R. China

**Keywords:** Biological sciences, Male reproductive endocrinology

## Abstract

The human endogenous retrovirus W envelope protein syncytin-1 (syncytin-a in mus) can be involved in fetal development and tumorigenesis. The role of syncytin-1 in male infertility has not been identified. In this study, the prostatic epithelial-specific syncytin-a knockout (syna CKO) mouse model was constructed. First, male infertility, abnormal testicular seminiferous tubule, and prostate gland were discovered in syna CKO mice. Then, irregular sperm motility, morphology, and MMAF-associated gene expression (AKAP3, SPAG6) were detected in syna CKO mice. Increased prostaglandin E2 (PGE2) and prolactin (PRL) modulate the lipid metabolism of the testis in syna CKO mice. Syna CKO with inhibitor or activator treatment identified that syna knockout could inhibit the PI3K/AKT/mTOR pathway, Ca^2+^ levels, and elevate caspase 3 expression. Therefore, we propose a hypothesis that syna deficiency exacerbates testicular lipid accumulation and apoptosis by suppressing the PI3K/AKT/mTOR and Ca^2+^ pathway, ultimately leading to spermatogenic dysfunction and male infertility.

## Highlights

• Syna CKO leads to male infertility and abnormal testes
• Syna deficiency impairs sperm motility, morphology, and the expression of MMAF genes
• Elevated PGE2 and PRL disrupt testicular lipid metabolism in syna CKO mice
• Syna CKO causes lipid accumulation and apoptosis via PI3K/AKT/mTOR and Ca^2+^

## Graphical abstract

**Figure:**
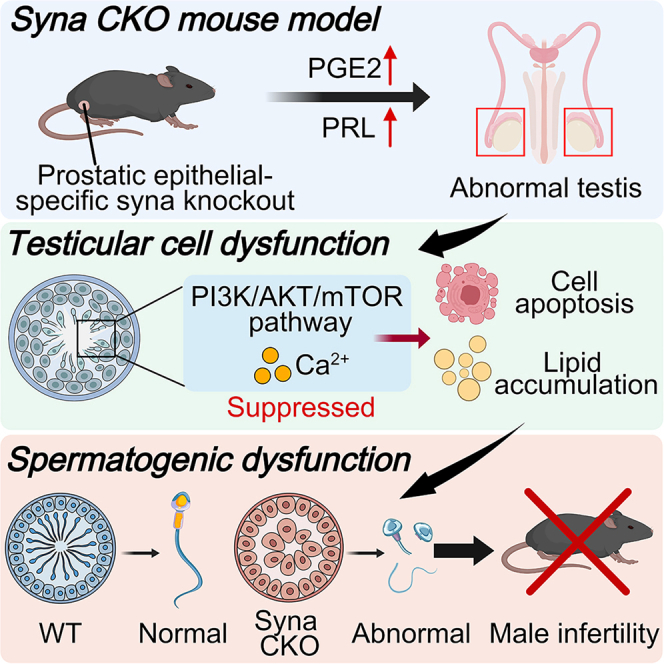

## Introduction

Infertility is a reproductive system disorder. The World Health Organization (WHO) defined it as couples of childbearing age failing to achieve a normal pregnancy after 12 months of regular, unprotected sexual intercourse.^1^ Recent epidemiological data revealed that approximately 186 million individuals worldwide are affected by this condition, with a concerning trend toward an earlier age of onset.^2^ About 30–50 percent of infertility cases are caused by the male factor, male infertility.^3^ Nowadays, the therapy methods for male infertility are mainly lifestyle optimization, medication, and surgery.^3^^,^^4^ There are many factors contributing to male reproductive disorders,^5^^,^^6^^,^^7^ such as genetic factors, endocrine factors, immunological factors, reproductive tract infections, sexual dysfunction, vas deferens obstruction, and testis spermatogenic dysfunction. Of these, genetic factors account for more than 15 percent of male infertility. The molecular mechanisms of male infertility still need further research.

Human endogenous retroviruses (HERVs) are derived from extinct exogenous retroviruses through multiple infections and integration during primate evolution.^8^ They account for approximately 8% of human DNA.^9^ Based on the type of tRNA recognized by the primer binding site, HERV can be divided into at least 31 families, including HERV-W,^10^ HERV-K,^11^ HERV-T,^12^ HERV-F,^13^ HERV-E,^14^ and so on. The abnormal expression of HERV is often associated with the development of malignancies and diseases, such as colorectal cancer,^15^ breast cancer,^16^ multiple sclerosis,^17^ neurodegenerative disorders,^18^ and so on. Most of the genes in the HERV family are silenced, but some genes still maintain their functions. HERV-W envelope protein (syncytin-1 in humans) is a highly fusogenic membrane glycoprotein that plays an important role in the process of receptor recognition and membrane fusion.^19^^,^^20^^,^^21^ Its abnormal expression was significantly associated with urothelial cell carcinoma of the bladder,^22^ non-small cell lung cancer,^23^ preeclampsia,^24^ and so on. Studies have found that the expression of syncytin-1 is significantly lower in samples with weak spermatozoa, oligozoospermia, and weak oligozoospermia.^25^ However, the mechanisms of syncytin-1 involved in male infertility have not been studied.

Mus Syncytin-a/b (syna/b), as syncytin-1/2 homologous genes, are a pair of fusogenic placenta-specific murine envelope genes from endogenous retroviruses. They are highly conserved and important for murine syncytiotrophoblast formation.^26^ The homozygous syna null mice have embryonic lethality, which die *in utero* between 11.5 and 13.5 days of gestation.^27^ However, there are no reports detailing the specific mechanisms of syna gene deletion in male infertility. Based on this, we pre-constructed the prostatic epithelial-specific syna conditional knockout (CKO) mouse model. We found that the prostatic epithelial-specific syncytin-a knockout (syna CKO) male mice have lower fertility than wild-type mice. And we further investigated sperm abnormalities, the abnormal development of the testes and prostates, and the potential molecular mechanisms for syna in male infertility. The aim is to clarify whether syncytin-1 could serve as an emerging biological marker and a potential therapeutic target for male infertility.

## Results

### Generation and identification of syna CKO male mice

**Figure 1:**
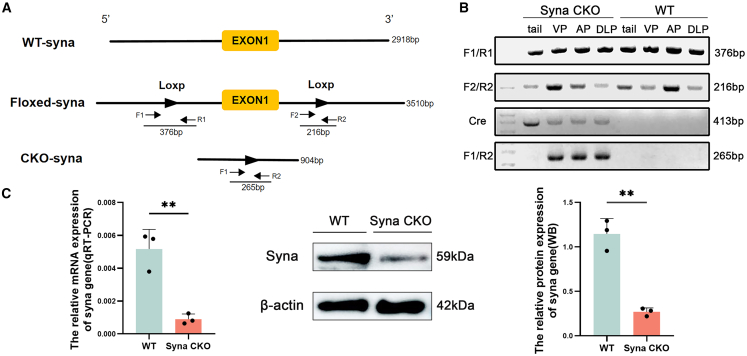
Generation and identification of syna conditional knockout mice (A) The syna gene CKO strategy. (B) Mouse genotypes were determined by PCR of DNA isolated from prostate and tail tissues. (C) The relative mRNA and protein expressions of the syna gene by qRT-PCR and WB. Data are presented as mean ± SD of *n* = 3 biologically independent experiments. Mean ± standard error is displayed in dot plots and bar charts. ∗∗*p* < 0.01 by Student’s *t* test.

The syna CKO male mouse model was constructed using the CRISPR-Cas9 system, selecting the ATG start codon in exon 1 and the TAG stop codon in exon 1 as the target sites (Figure 1A). The probasin gene promoter is specifically expressed in the prostate.^28^ According to the breeding program, syna CKO male mice were acquired with probasin promoter-driven cre (pbsn-cre) male mice (Figure S1). The prostatic epithelial-specific syna knockout mouse model using the pbsn-cre (pbsn-cre, syna^f/f^; named syna CKO VS syna^f/f^; named WT) was confirmed by PCR (Figure 1B). The presence of both F1/R1 (376bp) and F2/R2 (216bp) bands proves that the mice are syna^f/f^ genotype. There is a cre band (413bp) in syna CKO mice and not in WT mice. There is a 265bp amplification fragment in the prostate but not in the tail of the syna-CKO mice. Gene sequencing again verified the genotypes (Figure S2). qRT-PCR and WB demonstrated that the mRNA and protein expression of syna was decreased significantly in the syna CKO male mice (*p* < 0.01) (Figure 1C). These results demonstrated that the prostate-specific syna knockout mouse model was constructed successfully.

### Syna absence affects male fertility and testis/prostate development

**Figure 2:**
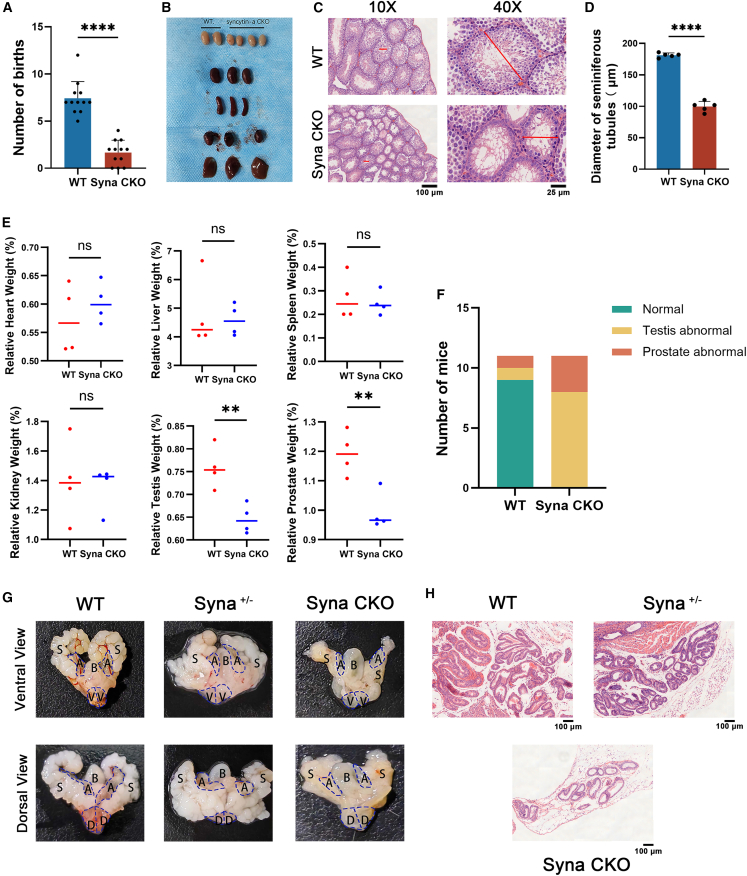
Syna CKO impaired fertility and development in male mice (A) Comparison of litter size between WT male mice (plug *N* = 12) and syna male mice (plug *N* = 12). Data are presented as mean ± SD of *n* = 12 biologically independent experiments. (B) A picture of the heart, liver, spleen, kidney, and testis tissues. (C) HE staining of the testis. The red straight line represents the length of the testicular seminiferous tubule diameters. Scale bars, 100 μm and 25 μm. (D) A statistical plot of the diameter of seminiferous tubules. Data are presented as mean ± SD of *n* = 5 biologically independent experiments. (E) Comparison of heart, liver, spleen, kidney, testis, and prostate weights in 4-month-old WT and syna CKO male mice. Data are presented as mean ± SD of *n* = 4 biologically independent experiments. (F) The percentage of testis and prostate abnormalities in syna CKO mice. (G) In ventral view and dorsal view, different morphologies of prostate tissue in three different genotypes, WT, syna^+/−^ (pbsn cre, syna^f/-^), and syna CKO. A: anterior prostate, B: bladder, D: dorsal-lateral prostate, S: seminal vesicle, and V: ventral prostate. (H) HE staining of the prostate from WT male mice, syna^+/-^ male mice, and syna CKO male mice. Scale bars, 100 μm. Mean ± standard error is displayed in dot plots and bar charts. ∗∗*p* < 0.01 and ∗∗∗∗*p* < 0.0001 by Student’s *t* test.

The fertility test was separately conducted in six groups of WT and syna CKO male mice, with 12 plug counts recorded per group. The results indicated that there were significantly fewer litters in the syna CKO group than in the WT group (*p* < 0.0001) (Figure 2A). The gross and histological levels were then analyzed in syna CKO and WT male mice. The testes were smaller in syna CKO mice (Figure 2B). The anterior prostate (AP), ventral prostate (VP), and dorsal-lateral prostate (DLP) of the prostate are progressively smaller in the WT, syna^+/−,^ and syna CKO mice (Figure 2G). Similarly, the weight of the testes (*p* < 0.01) and prostates (*p* < 0.01) in syna CKO mice was significantly lower than in WT mice. However, there were no statistical differences in the heart, liver, spleen, and kidney between 4-month-old syna CKO and WT mice (*p* > 0.05) (Figure 2E). Based on these, we analyzed the percentage of testis and prostate abnormalities in syna CKO mice. The results showed that the syna CKO does not necessarily affect both testis and prostate development, but at least one of them will certainly develop abnormally (Figure 2F). In WT mice, testicular spermatogenic epithelial cells were tightly arranged, with intact basement membranes and clear boundaries. A large number of spermatogonia in the lumen of the seminiferous tubules and capillaries in the testicular interstitium. However, the testicular spermatogenic epithelial cells were loosely arranged in syna CKO mice. The testicular seminiferous tubule diameters were smaller (*p* < 0.0001), with fewer spermatocytes in the lumen of the seminiferous tubules, altered morphology of spermatogonia, a decrease in the number of cell layers in the wall of the tubules, and a decline in the number of Leydig cells and capillaries in the interstitium (Figures 2C and 2D). Consistent with testis defects, HE staining of the cauda epididymis revealed that the lumens of WT male mice were filled with abundant, dense, and well-aligned sperm. In contrast, although the total amount of sperm in the syna CKO mice appeared substantial, the sperm were loosely arranged and disorganized, accompanied by the accumulation of abnormal cellular debris (Figure S3). The HE staining of prostate tissue showed that the prostate was smaller in syna CKO mice (Figure 2H). Immunofluorescence (IF) staining of prostate sections showed that the expression of syncytin-1 was significantly lower in syna CKO male mice than in WT male mice, and the prostate epithelial secretory cells were loosely arranged (Figure S4). All in all, syna deficiency causes decreased fertility and abnormal testes/prostate development.

### Syna CKO leads to low sperm motility and abnormal sperm morphology

**Figure 3:**
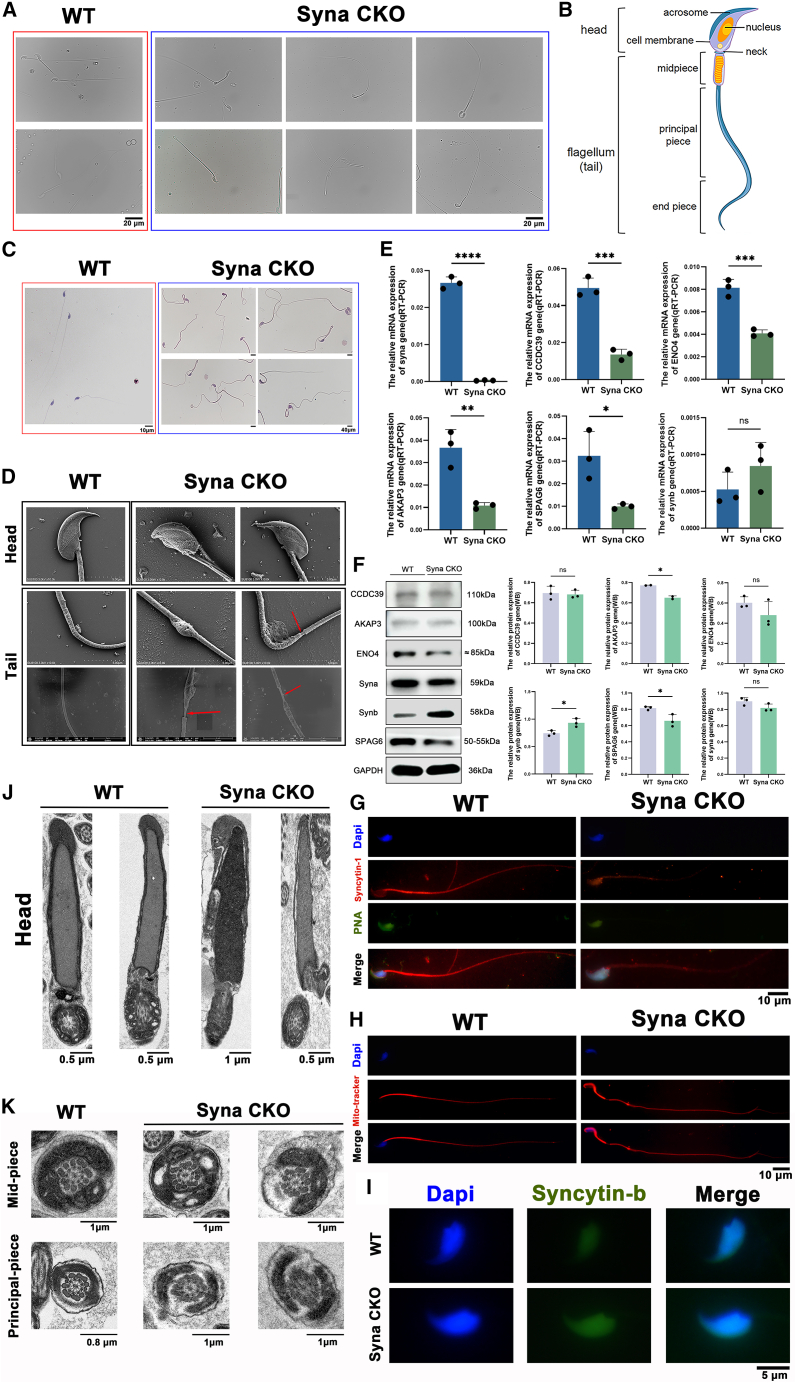
Syna CKO disrupts sperm morphology and ultrastructure (A) Sperm morphology analysis by the electron microscope. Scale bars, 20 μm. (B) Schematic diagram of normal mouse sperm structure. (C) HE staining of sperm. Scale bars, 10 μm and 40 μm. (D) Scanning electron micrographs of the sperm head and tail of WT and syna CKO mice. Scale bars, 4 μm and 5 μm. (E) The relative mRNA expressions of ENO4, AKAP3, CCDC39, SPAG6, syna, and synb genes. GAPDH was used as an internal control. Data are presented as mean ± SD of *n* = 3 biologically independent experiments. (F) The relative protein expressions of ENO4, AKAP3, CCDC39, SPAG6, syna, and synb genes. GAPDH was used as an internal control. Data are presented as mean ± SD of *n* = 3 biologically independent experiments. (G) Sperm immunofluorescence for syncytin-1 (red), PNA (green), and Dapi (blue). Scale bars, 10 μm. (H) Sperm immunofluorescence for mito-tracker (red) and Dapi (blue). Scale bars, 10 μm. (I) Sperm immunofluorescence for syncytin-b (green) and Dapi (blue). Scale bars, 5 μm. (J) Transmission electron micrographs of the sperm head. Scale bars, 0.5 μm and 1 μm. (K) Transmission electron micrographs of the sperm midpiece and principal piece. Scale bars, 0.8 μm and 1 μm. Mean ± standard error is displayed in dot plots and bar charts. ∗∗∗∗*p* < 0.0001, ∗∗∗*p* < 0.001, ∗∗*p* < 0.01, and ∗*p* < 0.05 by Student’s *t* test.

Normal mouse sperm is structured into a head, neck, midpiece, principal piece, and end piece.^29^ The head of the sperm is curved and sickle-shaped with a smooth surface. The acrosome is located on the head (Figure 3B). The sperm motility of syna CKO male mice is reduced, and the movement direction is irregular (Video S2) compared to the WT male mice (Video S1). The morphology has abnormal changes. The sperm head defects are mainly round heads, double heads, or headless. The main defects of the neck are an unusually slender neck or one that is bent at an angle. The end piece defects are mainly short, broken, or curled tails (Figure 3A). The HE staining of sperm also proved the results (Figure 3C). The SEM results further showed that the sperm of WT mice had normal structures, smooth sickle-shaped heads, and continuous flagella. But the syna CKO sperm heads turned rounded or misshapen. The fibrous structure of the flagellum was also abnormal (Figure 3D).

Video S1. The video of sperm motility in WT mice

Video S2. The video of sperm motility in syna CKO mice

The multiple morphological abnormalities of the flagella (MMAF) phenotypes represent a well-characterized etiology of male infertility, clinically defined by flagellar defects (shortened, absent, bent, coiled, or irregular morphology) accompanied by reduced motility.^30^ Studies have found that MMAF is associated with multiple gene mutations, such as DNAH1,^30^ DNAH2,^31^ DNAH6,^32^ DNAH17,^33^ CFAP61,^34^ CFAP65,^35^ CFAP69,^36^ ARMC2,^37^ AK7,^38^ and so forth. The above abnormal morphologies are very similar to the MMAF phenotypes. We verified the relative mRNA and protein expression of MMAF-related genes in syna CKO male mice. The results showed the relative mRNA expression of syna (*p* < 0.0001), CCDC39 (*p* < 0.001), ENO4 (*p* < 0.001), AKAP4 (*p* < 0.001), SPAG16 (*p* < 0.001), AKAP3 (*p* < 0.01), SPEF2 (*p* < 0.01), CCDC40 (*p* < 0.01), CFAP43 (*p* < 0.01), CFAP44 (*p* < 0.01), CFAP69 (*p* < 0.01), SPAG6 (*p* < 0.05) and DNAH1 (*p* < 0.05) was significant differences. However, the expressions of synb and HYDIN were not statistically significant (Figures 3E and S5). CCDC39, AKAP3, ENO4, SPAG6, syna, and synb genes were further evaluated at the protein level. Moreover, the relative protein expressions of AKAP3 and SPAG6 were lower in syna CKO male mice than in WT (*p* < 0.05), but the synb expression was higher in syna CKO male mice (*p* < 0.05) (Figure 3F). The IF also validated the results of syna and synb. The expression of syncytin-1 in the principal piece also decreased (Figure 3G). And syncytin-2 is expressed in the sperm’s head. Its expression is higher in syna CKO sperm (Figure 3I). Thus, low expression of the syna gene might cause the MMAF phenotype, which affects spermatogenesis and causes male infertility.

### Syna absence causes sperm ultrastructure abnormality

The basic structure of the sperm flagellum is the axoneme, which contains nine peripheral microtubule doublets, surrounding a central pair of microtubules (9 + 2 pattern).^39^ To observe the effect of syna CKO on the sperm, we examined the ultrastructure of the sperm using TEM analysis. The results showed that the morphology of the WT sperm head was normal, the nuclear density was uniform, the acrosome structure was intact, and there was a clear boundary between the nucleus and the acrosome. In contrast, the morphology of the syna CKO sperm head had abnormalities, with irregular structure, broken or missing acrosome, and blurred boundaries between acrosome and nuclei (Figure 3J). The IF results confirmed this. Peanut agglutinin (PNA) is not expressed in the acrosome but in the nucleus (Figure 3G). In the syna CKO sperm flagella, the mitochondrial sheath appeared hollow, fuzzy, and discontinuous in the midpiece. The outer dense fibers (ODFs) appeared disordered and deformed, and the fibrous sheath was incomplete or broken in the principal piece (Figure 3K). Sperm staining with Mito-Tracker Red CMXRos results also showed one break location in the connection between the midpiece and principal piece (Figure 3H). All of these data suggested that mitochondria and ODFs might be destroyed in the connection between the midpiece and principal piece. Thus, the absence of syna causes sperm ultrastructure abnormalities, which may be one of the reasons for male infertility.

### Syna absence causes male infertility by regulating the levels of PRL and PGE2 hormones

**Figure 4:**
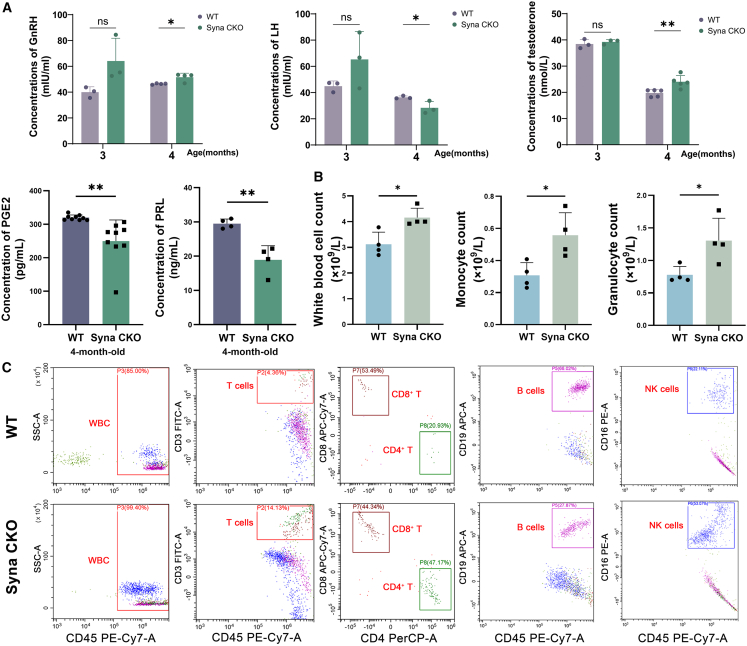
Syna CKO modulates immune response by regulating the levels of PRL and PGE2 hormones (A) The concentration changes of GnRH, LH, testosterone (T), PGE2, and PRL. Data are presented as mean ± SD. (B) The counts of white blood cells, monocytes, and granulocytes. Data are presented as mean ± SD of *n* = 4 biologically independent experiments and *n* = 3 technical replicates. (C) Flow cytometric analysis of immune cells between WT and syna CKO male peripheral blood. Mean ± standard error is displayed in dot plots and bar charts. ∗∗*p* < 0.01 and ∗*p* < 0.05 by Student’s *t* test.

Mouse testosterone (T), gonadotropin-releasing hormone (GnRH), luteinizing hormone (LH), prostaglandin E2 (PGE2), and prolactin (PRL) were detected to explore the hormonal reaction in the fertility defects of syna CKO mice. The concentrations of GnRH (*p* < 0.05) and testosterone (T) (*p* < 0.01) increased, and the concentrations of LH (*p* < 0.05), PGE2 (*p* < 0.01), and PRL (*p* < 0.01) decreased in 4-month-old syna CKO male mice (Figure 4A). There was no significant difference in testosterone (T), GnRH, and LH concentrations between 3-month-old syna CKO and WT male mice. PRL and PGE2 have an important impact on modulating the immune response.^40^^,^^41^ Based on this, the results of the mouse routine blood count test revealed significantly higher white blood cell counts (*p* < 0.05), monocyte counts (*p* < 0.05), granulocyte counts (*p* < 0.05), and mean corpuscular hemoglobin concentration (MCHC) (*p* < 0.05) in syna CKO male mice than in WT male mice. But the counts of lymphocyte, hemoglobin, red blood cell, and blood platelet showed no significant difference (Figures 4B and S6). Flow cytometry was performed to further analyze its effect on immune deficiency. In syna CKO male mice, all the counts of white blood cells, CD45^+^CD3^+^ T cells, CD45^+^CD3^+^CD4^+^ T cells, and CD45^+^CD16^+^ NK cells increased, but the counts of CD45^+^CD3^+^CD8^+^ T cells and CD45^+^CD19^+^ B cells decreased (Figure 4C). Therefore, syna CKO may cause a decrease in PRL and PGE2 hormone concentrations, impair immune response, and further impact male reproduction.

### Syna CKO could inhibit the Ca^2+^ and PI3K/AKT/mTOR signaling pathway to regulate lipid metabolism, leading to male infertility

**Figure 5:**
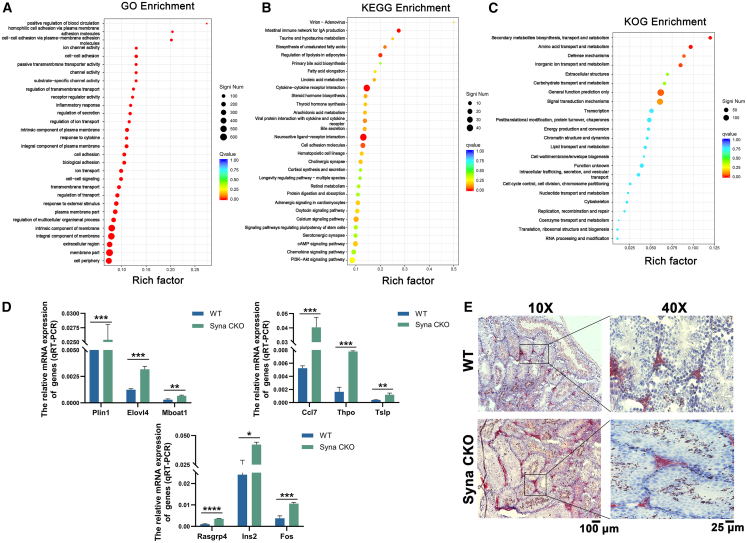
RNA-seq and lipid metabolism analysis of syna CKO testis tissues (A) Bubble diagram of GO enrichment analysis. (B) Bubble diagram of KEGG pathway enrichment analysis. (C) Bubble diagram of KOG enrichment. (D) The relative mRNA expression of lipid metabolism-related genes (plin1, elovl4, and mboat1), cytokine-cytokine receptor interactions-related genes (ccl7, thpo, and tslp), and signaling pathway-related genes (rasgrp4, ins2, and fos). Data are presented as mean ± SD of *n* = 3 biologically independent experiments. (E) The images of oil red staining between WT and syna CKO male mice. Scale bars, 100 μm and 25 μm. Mean ± standard error is displayed in dot plots and bar charts. ∗∗∗∗*p* < 0.0001, ∗∗∗*p* < 0.001, ∗∗*p* < 0.01, and ∗*p* < 0.05 by Student’s *t* test.

To explore the specific molecular mechanisms of syna to regulate male reproduction, we compared the differences in transcriptional profiles between WT and CKO male mice by RNA-Seq analysis. The heatmap revealed the distance between WT and syna CKO male mice (Figure S7A). The volcano map showed that a total of 174 genes were differentially expressed, including 131 up-regulated genes and 43 down-regulated genes (Figure S7B). KEGG enrichment analysis predicted 23 up-regulated genes and 9 down-regulated genes that may be involved in regulating signaling pathways. qRT-PCR was carried out to verify the predicted abnormal genes in KEGG. The relative mRNA expressions of the rasgrp4 (*p* < 0.0001), rpe65 (*p* < 0.0001), ccl7 (*p* < 0.001), elovl4 (*p* < 0.001), fos (*p* < 0.001), olfr1420 (*p* < 0.001), olfr1509 (*p* < 0.001), plin1 (*p* < 0.001), thpo (*p* < 0.001), eif3j2 (*p* < 0.01), inhbc (*p* < 0.01), mboat 1 (*p* < 0.01), olfr981 (*p* < 0.01), pigr (*p* < 0.01), ren1 (*p* < 0.01), tslp (*p* < 0.01), CCR9 (*p* < 0.01), cckar (*p* < 0.05), ins2 (*p* < 0.05), trpc3 (*p* < 0.05), and hist1h2br (*p* < 0.05) genes were increased in syna CKO compared to WT. But the relative mRNA expressions of the col5a3 (*p* < 0.001), hsd3b7 (*p* < 0.01), kcnj12 (*p* < 0.01), lgr5 (*p* < 0.05), hist2h3c1 (*p* < 0.05), and olfr1336 (*p* < 0.05) genes were decreased. No significant difference was found in the expressions of il18r1, olfr273, and alg6 genes between groups (Figure 5D; Figure S8). 17 up-regulated genes and 4 down-regulated genes were found, almost consistent with the predicted results.

To study the role of syna in various biological processes, molecular functions, and pathways. RNA-seq results were analyzed for Gene Ontology (GO) gene function classification, KEGG pathway classification, and KOG function classification (Figures S7C–S7E). GO enrichment between WT and syna CKO male mice mainly included substrate-specific channel activity, inflammatory response, response to cytokine, plasma membrane part, and regulation of the multicellular organismal process et al. (Figure 5A). KEGG pathway analysis revealed that syna could affect cell proliferation, survival, or differentiation functions through lipid metabolism-related pathways, PI3K-AKT signaling pathway, cAMP signaling pathway, calcium signaling pathway, and so on (Figure 5B). KOG enrichment analysis revealed that syna is mainly related to secondary metabolites biosynthesis, amino acid transport and metabolism, and inorganic ion transport and metabolism, suggesting that syna could be involved in metabolic regulation, signaling, and other processes (Figure 5C). RNA-seq predicted that the plin1, elovl4, and mboat1 are associated with lipid metabolism. The ccl7, thpo, and tslp may participate in cytokine-cytokine receptor interactions and the interactions between viral proteins and cytokines and their receptors. Tslp and thpo are involved in the JAK/STAT signaling pathway, while rasgrp4, ins2, and fos play roles in the MAPK and PI3K/AKT/mTOR signaling pathways. Their relative mRNA expressions were significantly higher in syna CKO male mice than in WT (Figure 5D). Oil red O staining revealed excessive lipid accumulation in syna CKO testes, primarily in the testicular interstitium (Figure 5E). This is consistent with the validation results of qRT-PCR. In conclusion, these results suggest that syna CKO may contribute to male infertility by affecting lipid metabolism.

**Figure 6:**
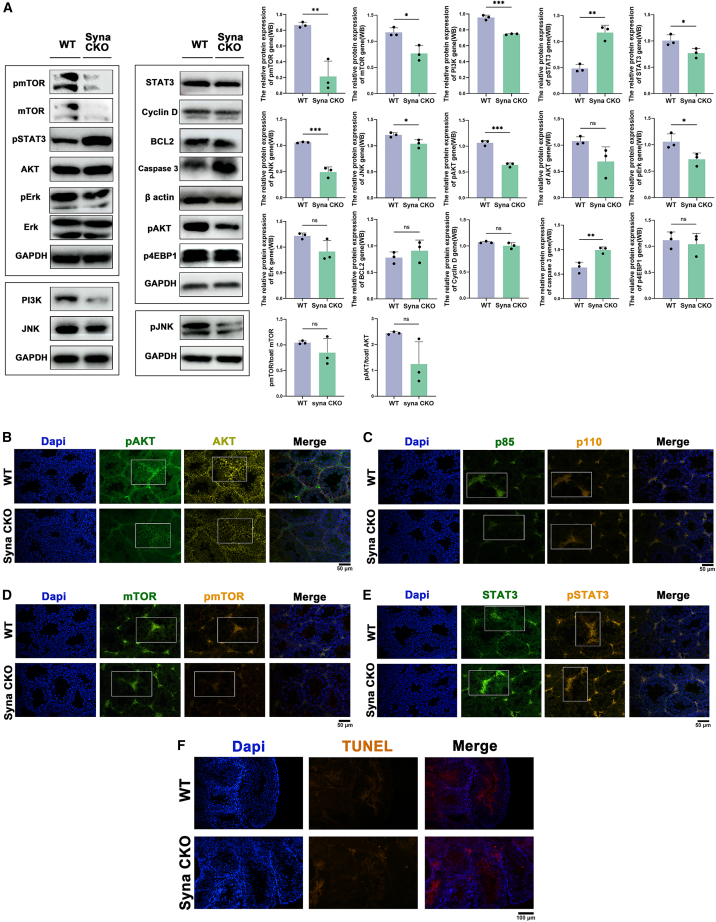
Syna CKO may affect infertility by inhibiting the PI3K/AKT/mTOR signaling pathway (A) The relative protein expression of different genes by WB. Data are presented as mean ± SD of *n* = 3 biologically independent experiments. (B–E) Testis tissues from WT and syna CKO male mice were stained with anti-AKT (yellow)/pAKT (green), anti-PI3K p85 (green)/p110 (orange), anti-mTOR (green)/pmTOR (orange), and anti-STAT3 (green)/pSTAT3 (orange), and DAPI (blue). Scale bars, 50 μm. (F) Testis TUNEL staining of WT and syna CKO male mice was stained with DAPI (blue) and TUNEL (orange). Mean ± standard error is displayed in dot plots and bar charts. ∗∗∗*p* < 0.001, ∗∗*p* < 0.01, and ∗*p* < 0.05 by Student’s *t* test.

**Figure 7:**
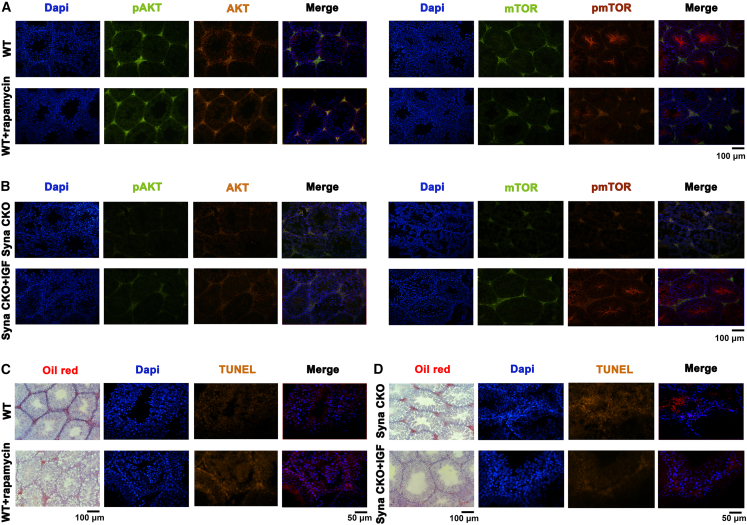
Activation of PI3K/AKT/mTOR signaling pathway rescues apoptotic defects in Syna CKO mice (A and B) Testis tissues from WT mice treated with or without the mTOR inhibitor rapamycin (A) and from Syna CKO mice treated with or without the PI3K activator IGF-1 (B). Tissues were stained with anti-AKT (orange)/pAKT (green), anti-mTOR (green)/pmTOR (red), and Dapi (blue). Scale bars, 100 μm. (C and D) The oil red O staining and TUNEL staining (orange) show lipid accumulation and apoptotic cells in testis sections from WT mice treated with or without rapamycin and syna CKO mice treated with or without IGF-1. Scale bars, 50 μm and 100 μm.

Combined with the KEGG bubble map co-analysis, the protein level further verifies the signal pathway (Figure 6A). The results showed that the ratios of pmTOR/total mTOR (*p* = 0.2905) and pAKT/total AKT (*p* = 0.0746) decreased, even though no statistical significance. The relative protein expressions of pERK (*p* < 0.05), pJNK (*p* < 0.001), PI3K (*p* < 0.001), pAKT (*p* < 0.001), and pmTOR (*p* < 0.01) were all decreased in syna CKO male mice. However, the expression of pSTAT3 increased (*p* < 0.01). IF also proved this result (Figures 6B–6E). In addition, data in Figure 6A showed that the expression of caspase 3 (*p* < 0.01) increased in the syna CKO group, but there was no statistical significance of the BCL2 and cyclin D genes. Consistent with these molecular findings, TUNEL staining results showed that the apoptosis of syna CKO testis significantly increased compared to the WT mice, and the apoptotic signals were predominantly localized to the testicular interstitium and adjacent to spermatozoa (Figure 6F). After treatment with rapamycin (mTOR inhibitor) in WT mice, testis IF results showed that the pmTOR and mTOR levels decreased compared to WT-untreated mice, while the pAKT and AKT levels increased. In contrast, IGF-1 (PI3K activator) treatment resulted in the upregulation of pmTOR, mTOR, pAKT, and AKT expression in syna CKO male mice (Figures 7A and 7B). Meanwhile, the results of oil red O staining and TUNEL staining also increased (Figures 7C and 7D). Collectively, these results suggest that syna CKO could lead to male infertility by inhibiting the PI3K/AKT/mTOR signal pathway, increasing lipid accumulation, and apoptosis.

**Figure 8:**
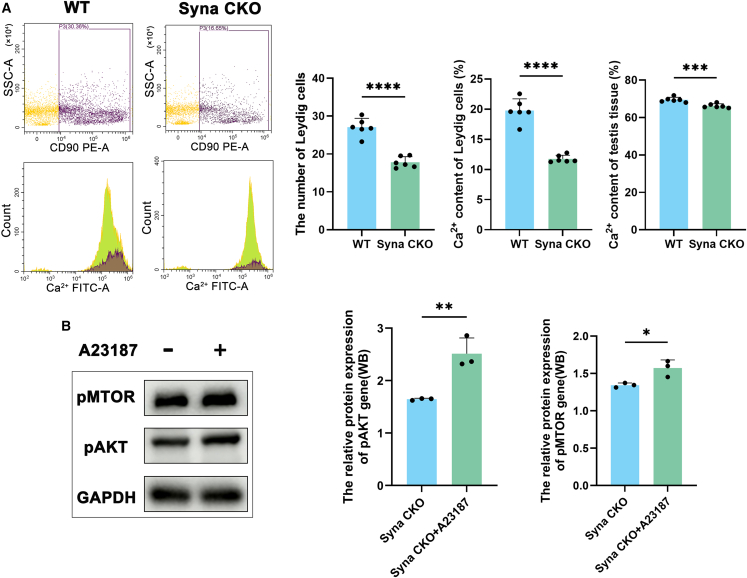
Syna CKO may affect infertility by regulating the PI3K/AKT/mTOR signaling pathway via Ca^2+^ (A) Flow cytometric analysis of Ca^2+^ and Leydig cells between WT and syna CKO testis tissues. Data are presented as mean ± SD of *n* = 6 biologically independent experiments and *n* = 3 technical replicates. (B) The relative protein expression of pmTOR and pAKT in the syna CKO group treated with or without the A23187. Data are presented as mean ± SD of *n* = 3 biologically independent experiments. Mean ± standard error is displayed in dot plots and bar charts. ∗∗∗∗*p* < 0.0001, ∗∗∗*p* < 0.001, ∗∗*p* < 0.01, and ∗*p* < 0.05 by Student’s *t* test.

Moreover, flow cytometry results showed that total Ca^2+^ content in testis tissues (*p* < 0.001), Leydig cell numbers (*p* < 0.0001), and Ca^2+^ content in Leydig cells (*p* < 0.0001) of syna CKO male mice were significantly lower than those of WT (Figure 8A). To investigate whether this Ca^2+^ deficiency impairs downstream signaling, the syna cre mouse embryonic fibroblasts (MEFs) cells were treated with calcium ionophore A23187. The WB results revealed that A23187 significantly upregulated the protein levels of pAKT (*p* < 0.01) and pmTOR (*p* < 0.05) compared to the syna CKO group (Figure 8B). Taken together, syna CKO may affect infertility by regulating lipid metabolism and apoptosis mediated by the Ca^2+^ and PI3K/AKT/mTOR signaling pathway.

## Discussion

Previous studies have established a correlation between syncytin-1 deficiency and male reproductive disorders. Gizem et al. demonstrated significantly reduced syncytin-1 expression in clinical cases of asthenozoospermia, oligozoospermia, and oligoasthenozoospermia.^25^ Notably, Anne et al. reported embryonic lethality in homozygous syna null murine models,^27^ underscoring its critical role in developmental biology. To further investigate the reproductive-specific function of syncytin-1 in males, the prostatic epithelial-specific syna CKO mouse model was constructed. Studies found that the abnormal development or dysfunction of the testis, epididymis, and prostate are all closely associated with male infertility.^42^^,^^43^^,^^44^ Data in Figures 2A–2D and S3 revealed that reproductive impairment was discovered in syna CKO male mice, characterized by significant reductions in testis and prostate organ size. HE staining showed that the testicular seminiferous tubules, cauda epididymis, and prostate gland were abnormal. These structural abnormalities were strongly associated with the male infertility phenotype. Although distinct testis developmental abnormalities were observed in prostate-specific syna knockout mice, this phenotype may be attributed to baseline non-specific deletion in the testis. In short, our findings suggested that prostate-specific syna CKO disrupts male reproductive development and function.

Syna CKO sperm revealed significant ultrastructural and functional abnormalities. The sperm had severe head abnormalities, including rounded, doubled, or headless. The ultrastructure of the head features irregular nuclear contours and loss of structural demarcation between the nuclear and acrosome. IF analysis showed a complete absence of PNA polarized acrosomal localization. These findings indicate that syna deficiency may impair the critical fertilization processes. Data in Figure 3 showed that the sperm flagella were coiled or absent, which was associated with MMAF phenotypes. The relative mRNA expression of MMAF-related genes showed differences in syna CKO testes, including CCDC39/40, ENO4, AKAP3/4, SPAG6/16, SPEF2, CFAP43/44/69, and DNAH1. The relative protein expression of AKAP3, synb, and SPAG6 genes also significantly differed. All in all, these collectively demonstrate that syncytin-1 deficiency causes sperm abnormality, ultimately leading to reproductive failure.

Abnormalities in the testis-prostate axis could be affected by long-distance transport of hormones, so we measured the concentrations of androgenic reproduction-related hormones. Results in Figure 4A showed that the concentrations of PRL (*p* < 0.01) and PGE2 (*p* < 0.01) were decreased. Previous studies demonstrated that PRL triggers the production of PGE2, which binds to EP2/4 receptors in the cell membranes of testis tissue.^40^^,^^41^^,^^45^ Therefore, syna knockout might affect spermatogenesis by decreasing PRL/PGE2 concentration. PGE2 can also influence the immune response. The results of routine blood tests and flow cytometry confirmed this. In addition, we speculate that the increased concentration of testosterone (T) may be due to the negative feedback effects of sperm abnormalities. The reduction in PGE2 (a physiological inhibitor of Leydig cells) removes the suppression on steroidogenesis, allowing the remaining Leydig cells to autonomously overproduce testosterone (T), potentially supported by other signals from the increased immune cells.^46^ Consistently, in this manuscript, the elevated testosterone (T) and decreased LH were detected in syna CKO mice. In conclusion, syna CKO may cause male infertility by decreasing the concentration of PRL/PGE2 and regulating the immune response.

Syna CKO may contribute to the development of male infertility by affecting apoptosis, lipid metabolism-related gene expression, and signaling pathways. KEGG enrichment analysis predicted 23 up-regulated genes and 9 down-regulated genes. All these genes were detected by qRT-PCR. qRT-PCR results showed 17 up-regulated genes and 4 down-regulated genes, which were consistent with results predicted by RNA-Seq. Several genes involved in lipid metabolism (plin1, elovl4, mboat1), cytokine interactions (ccl7, thpo, tslp), PI3K/AKT, and MAPK signaling pathways (rasgrp4, ins2, fos) showed significant differences between the two groups. WB and IF results further confirmed that PI3K/AKT/mTOR and MAPK signaling pathway-related proteins were inhibited in CKO mice. The discrepancy between the total protein expression trend and the activation ratio may be attributed to the fact that syna CKO primarily affects the protein expression of these genes. WT mice treated with rapamycin inhibited the PI3K/AKT/mTOR pathway, syna CKO mice treated with IGF-1 rescued this pathway. Furthermore, the intracellular Ca^2+^ levels of testis tissues and testis Leydig cells were significantly reduced in syna CKO mice. Previous studies showed PI3K signaling regulates Ca^2+^ channel trafficking,^47^^,^^48^ and intracellular Ca^2+^ is vital for AKT phosphorylation.^49^^,^^50^^,^^51^ To determine whether calcium contributes to male infertility via the PI3K/AKT/mTOR pathway, the syna CKO MEF cells were treated with Ca^2+^ ionophore A23187. The results showed that A23187 significantly rescued the levels of pAKT and pmTOR. The expression of STAT3 was higher, which serves as a convergence point of multiple pathways, integrating extracellular signals to drive transcriptional reprogramming. These findings further confirmed that syna knockout could induce male infertility by inhibiting the PI3K/AKT/mTOR pathway.

**Figure 9:**
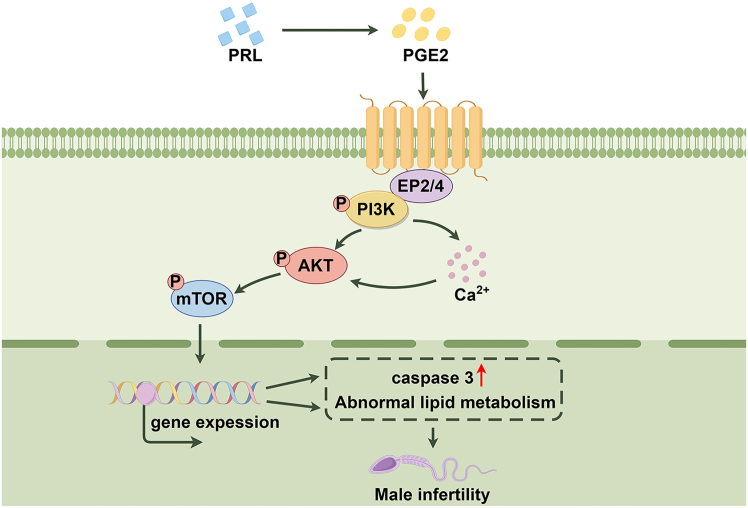
Diagram of the signaling pathway of syna CKO-induced male infertility PRL stimulates the production of PGE2, which binds to its receptors EP2/4 on the cell membrane. Activation of EP2/4 receptors leads to the phosphorylation of PI3K, subsequently activating the phosphorylation of AKT/mTOR and the release of Ca^2+^. The concentration of PRL/PGE2 decreased, and PI3K/AKT/mTOR signaling pathways were inhibited in syna CKO male mice, which caused caspase 3 overexpression and abnormal lipid metabolism, thereby contributing to male infertility.

The interplay between these pathways indicates that syna may regulate the fertility function by coordinating multiple intracellular signaling cascades. In previous publications, the inhibition of the PI3K/AKT signaling axis compromises mitochondrial function and disrupts metabolic homeostasis. This impairment typically leads to intracellular lipid overload due to defective fatty acid oxidation. The consequent lipotoxicity triggers fatal endoplasmic reticulum stress and activates the Bcl-2/Bax/Caspase-3 apoptotic pathway in testis cells, ultimately resulting in spermatogenic failure.^52^^,^^53^^,^^54^ In this manuscript, the expression of the lipid metabolism-related genes and caspase 3 was all upregulated in syna CKO mice. The oil red O staining and IF further demonstrated that apoptosis and lipid accumulation both occurred primarily in the testis interstitium. This excessive lipid deposition, compounded by the loss of AKT/mTOR-mediated survival signals, drives the sperm apoptotic process. The potential mechanisms underlying syna CKO-induced male infertility are summarized in Figure 9 as follows: First, syna CKO results in reduced circulating levels of PRL and PGE2. Second, the genetic ablation leads to the downregulation of Ca^2+^ and PI3K/AKT/mTOR signaling pathway, which triggers dual pathological effects: On the one hand, the activation of apoptotic pathways through the upregulation of the caspase 3 protein. On the other hand, the disruption of lipid metabolic processes. These syna CKO-mediated alterations in endocrine signaling, immune regulation, and cellular apoptosis/metabolic pathways collectively contribute to the pathogenesis of male infertility.

In conclusion, we found that syna deficiency was able to cause low fertility and abnormal morphology of the prostate, testis, and sperm in male mice. And syna deficiency disrupts PRL/PGE2 secretory homeostasis, thereby exacerbating testicular lipid accumulation and apoptosis by suppressing the Ca^2+^ and PI3K/AKT/mTOR signaling axis, ultimately leading to spermatogenic dysfunction and male infertility. Syna represents a potential biological marker and therapeutic target for male infertility.

### Limitations of the study

This study contains two main limitations. First, our results are primarily based on mouse models, necessitating clinical validation in human cohorts to confirm the diagnostic utility of syna. Second, the specific therapeutic application of syncytin-1 for treating male infertility requires further development. Future research will focus on human clinical validation and the optimization of syna-based interventions.

## Resource availability

### Lead contact

Further information and requests for resources and reagents should be directed to and will be fulfilled by the lead contact, H.L. Yu (yhonglian23@126.com).

### Materials availability

This study did not generate new unique reagents, and all materials are commercially available. The prostatic epithelial-specific syncytin-a conditional knockout (syna CKO) mouse model, and any additional analysis information in this study, are available by request to the lead contact.

### Data and code availability

• The raw and processed RNA-seq data reported in this paper have been deposited at GEO: GSE324927 and are publicly available as of the date of publication. Original western blot images and any other datasets reported in this paper will be shared by the lead contact upon request.
• This paper does not report original code.
• Any additional information required to reanalyze the data reported in this paper is available from the lead contact upon request.

## Acknowledgments

This research was funded by the 10.13039/501100007129Shandong Provincial Natural Science Foundation, grant no. ZR2020MH078 and ZR2020MH070, the Pudong New Area Health System Medical Discipline Development Project, grant no. 2024-G-D006, the Pudong New Area Health Commission Discipline Development Plan, grant no. PWZxk2025-05, and the College Students’ Innovative Entrepreneurial Training Plan Program of 10.13039/501100008853Jining Medical University, grant no. cx2024159z.

## Author contributions

Conceptualization, Z.K.Z. and H.L.Y.; methodology, Q.Q.W., Z.W.W., Z.P.L., and Q.B.; investigation, D.L.L., X.T.Y., Y.Z., and N.C.; writing – original draft, Q.Q.W.; writing – review and editing, Z.K.Z., H.L.Y., and Q.Q.W.; funding acquisition, Z.K.Z. and H.L.Y.; resources, Z.K.Z. and H.L.Y.; supervision, Z.K.Z. and H.L.Y.

## Declaration of interests

The authors declare no competing interests.

## STAR★Methods

### Key resources table

**Table:** 

REAGENT or RESOURCE	SOURCE	IDENTIFIER
**Antibodies**		

Syncytin-1	Bioss	Cat #bs-2962R; RRID: AB_11106881
ERVFRD1	Bioss	Cat #bs-15466R; RRID: AB_3720112
ENO4	Elabscience	Cat #E-AB-17910
CCDC39	Affinity	Cat #DF13893
SPAG6	Proteintech	Cat #12462-1-AP; RRID: AB_2194798
AKAP3	Proteintech	Cat #13907-1-AP; RRID: AB_2273887
GAPDH	Affinity	Cat #AF7021
β-actin	Affinity	Cat #AF7018
Phospho-AKT1	Abcepta	Cat #AP3434a; RRID: AB_1208350
Akt	Cell Signalling Technology	Cat #4691S; RRID: AB_915783
Phospho-STAT3	Abcam	Cat #ab76315; RRID: AB_1658549
STAT3	Abcam	Cat #ab119352; RRID: AB_10901752
p44/42 MAPK (Erk1/2)	Cell Signalling Technology	Cat #4695S; RRID: AB_390779
Phospho-p44/42 MAPK (pErk1/2)	Cell Signalling Technology	Cat #4370S; RRID: AB_2315112
Phospho-mTOR	Cell Signalling Technology	Cat #5536P; RRID: AB_10691552
mTOR	Cell Signalling Technology	Cat #2983P; RRID: AB_2105622
Phospho-4E-BP1	Cell Signalling Technology	Cat #2855P; RRID: AB_560835
Anti-Bcl-2	Abcam	Cat #ab196495; RRID: AB_2924862
Cleaved-Caspase 3	Affinity	Cat #AF7022
Cyclin D1	Boster	Cat #BM4272
Phospho-JNK1/2/3	Affinity	Cat #AF3318
JNK1/2/3	Affinity	Cat #AF6319
PI3 Kinase p110α	Omnimabs	Cat #OM644122
Syncytin-1	Biorbyt	Cat #orb100573; RRID: AB_2857960
β-Tubulin	Affiniity	Ca t#T0023
anti-rabbit IgG, HRP-linked antibody	Cell signaling technology	Cat #7074P2; RRID: AB_2099233
anti-mouse IgG, HRP-conjugated antibody	Abconal	Cat #AS003

**Chemicals, peptides, and recombinant proteins**		

TB Green Premix Ex Taq	Takara	Cat #RR820A
M-PER	Thermo scientific	Cat #QJ222436
4% (mass/vol) paraformaldehyde	Biosharp	Cat #BL539A
Rapamycin	MCE	Cat #HY-10219
IGF-1	Beyotime	Cat #P5502
PNA-FITC	Sigma	Cat #L7381
DAPI	Beyotime	Cat #P0131
Mito-Tracker Red CMXRos	Beyotime	Cat #C1049B
white blood cell marker CD45	Biolegend	Cat #147703,
T cell markers: CD3	Biolegend	Cat #100203,
T cell markers: CD8	Biolegend	Cat #100713
T cell markers: CD4	Biolegend	Cat #100433
B cell marker: CD19	Biolegend	Cat #115512
NK cell marker: CD16	Biolegend	Cat #158003
PE Anti-Mouse CD90 Antibody	Elabscience	Cat #E-AB-F1283D
Fluo 3-AM	Solarbio	Cat #F8840
Epoxy resin	Epon	Cat #812
Lenti-EF1α-Cre-P2A-EGFP-Puro	Beyotime	Cat #C4102
Calcium ionophore A23187	Glpbio	Cat #GC11200

**Critical commercial assays**		

Hematoxylin and eosin (HE) kit	Beyotime	Cat #C0105S
TUNEL staining kit	Glpbio	Cat #GK10041
Prime Script RT reagent Kit with gDNA Eraser	Takara	Cat #RR047A
Testosterone (T) ELISA kit	MEIMIAN	Cat #MM-0569M2
Gonadotropin-releasing hormone (GnRH) ELISA kit	MEIMIAN	Cat #MM-0506M2
Luteinizing hormone (LH) ELISA kit	MEIMIAN	Cat #MM-44039M2
Prostaglandin E2 (PGE2) ELISA kit	MEIMIAN	Cat #MM-0062M2
Prolactin (PRL) ELISA kit	MEIMIAN	Cat #MM-0553M2
Oil red O staining kit	Beyotime	Cat #C0157S

**Deposited data**		

RNA-seq	Sangon biotech, Shanghai, China	https://store.sangon.com/
Raw and processed RNA-seq data	This paper; Gene Expression Omnibus (GEO)	GSE324927

**Experimental models: Cell lines**		

E14 Syna^ff^ MEF cell	This paper.	N/A

**Experimental models: Organisms/strains**		

syna^f/-^ mouse	Cyagen biosciences, Suzhou	https://www.cyagen.com/
Pbsn-cre mouse	Cyagen biosciences, Suzhou	https://www.cyagen.com/

**Software and algorithms**		

Applied Biosystems QuantStudio 5 Real-Time PCR System	Thermo Fisher	https://www.thermofisher.com/
Image J software	ImageJ	https://imagej.nih.gov/ij
GraphPad Prism 10	GraphPad Software	https://www.graphpad.com/
Adobe Photoshop	Adobe Photoshop (2021)	RRID: SCR_014199
Adobe Illustrator	Adobe Illustrator (2021)	RRID: SCR_010279
CytExpert software (v.2.5)	Beckman Coulter	https://www.beckman.com/
FastQC (v.0.11.9)	Babraham Bioinformatics	RRID: SCR_014583
DESeq2	Love et al., 2014	RRID: SCR_000154
WGCNA	Langfelder and Horvath, 2008	RRID: SCR_003302
topGO	Alexa A, Rahnenführer J, 2010	RRID: SCR_014790
clusterProfiler	Yu et al., 2012	RRID: SCR_016884

### Experimental model and study participant details

#### Animals

The mouse syna gene (Gene ID: 214292) is located on chromosome 5, containing 6 exons. Exon 1 has a complete open reading frame. We targeted the ATG start codon in exon 1 and the TAG stop codon in exon 1, using the CRISPR-Cas9 system. The gRNA sequences are 5-TAACCTGGACAGTCTAACCCAGG-3 and 5-CCTTTGGTTGAGAAGCCCCCAGG-3. C57BL/6J heterozygous syna^f/-^ mouse model was constructed by Cyagen biosciences, Suzhou, China. Pbsn-cre is a transgenic mouse line with the prostate-specific expression of cre recombinase under the control of an ARR_2_PB promoter.^55^^,^^56^ It was provided by Cyagen biosciences, Suzhou, China. To obtain prostate-specific syna knockout male mice, we crossed syna^f/-^ female mice with Pbsn-cre male mice according to the scheme in Figure S1. We mainly used four methods to identify mouse genotypes: PCR amplification of genomic DNA from the tails and prostate tissues of the mice. The qRT-PCR experiments and WB were used to detect the expression of syna in prostate tissues. DNA sequencing for re-validation. The information on PCR primers used for genotyping is shown in Table S1. For homozygous mice, a 376bp fragment was obtained with F1/R1 paired primers, and a 216bp fragment with F2/R2 paired primers. When the syna was knocked out, a fragment of 413bp was obtained using the cre primer. A 265bp fragment was obtained using F1/R2 primers when syna was conditionally knocked out in the prostate. All mice were reared at the Animal Center of Jining Medical University, and all animal experiments were carried out according to the standards of the Animal Ethics Committee of Jining Medical University (JNMC-2024-DW-237).

Our research specifically investigates male infertility and spermatogenic dysfunction caused by syna deficiency, so this study focused exclusively on male mice.

#### Cell lines

Primary MEFs were isolated from E14 Syna^ff^ mouse embryos. MEFs were infected with Lenti-EF1α-Cre-P2A-EGFP-Puro (C4102, Beyotime) to generate syna-deficient cells. The primary MEFs were validated by embryo genotyping and subsequent PCR/Western blot confirmation of Cre-mediated recombination. All cells were routinely tested for mycoplasma contamination using a PCR-based detection kit and confirmed to be negative before use.

### Method details

#### Mouse fertility testing

The pbsn-cre, syna^flox/flox^ (syna CKO) male mice and their littermate control wild-type (WT) male mice aged 8-12 weeks. They were mated individually with four WT C57BL/6J female mice (8–16 weeks old, proven fertility). The number of vaginal plugs and pups was counted over 12 weeks, recording individual litters' numbers and birth dates.^57^

#### Quantitative real-time PCR (qRT-PCR)

Total RNA was isolated from the sperm, testis tissues, and prostate tissues of 4-month-old syna CKO mice or WT mice. The cDNA was acquired using the Prime Script RT reagent Kit with gDNA Eraser (RR047A, Takara). All qRT-PCR reactions were followed by TB Green Premix Ex Taq instructions (RR820A, Takara). The primer sequences used were all listed in Table S1. qRT-PCR was detected applied Biosystems QuantStudio 5 Real-Time PCR System (Thermo Fisher). GAPDH was used as an internal control. The levels of relative expression were calculated by the comparative C(T) method.

#### Western blotting (WB)

Protein was extracted using M-PER (QJ222436, Thermo scientific) from testes, prostate tissues, and ground with a cryo-mill (Scientz-48L). WB was performed following the same procedure as before [30]. Samples were incubated individually with primary antibodies, followed by incubation with the secondary antibody, anti-rabbit IgG, HRP-linked antibody (1:3000, 7074P2, Cell signaling technology), and anti-mouse IgG, HRP-conjugated antibody (AS003, Abconal). Detailed information about the primary antibodies was provided in Table S2. The β-actin and GAPDH served as internal standards.

#### Tissue collection and histological analysis

In 4-month-old WT male mice and syna CKO male mice, peripheral blood was first obtained for the mouse routine blood count test (YAN-305A, Yuyan). Then the testis, prostate, heart, liver, spleen, and kidney were obtained by dissection. The tissues were weighed and photographed. The testes and prostates were fixed in 4% (mass/vol) paraformaldehyde (BL539A, Biosharp), then embedded in OCT (Tissue-Tek O.C.T. compounds, Sakura), and 10 μm frozen sections were made (CM1950, Leica). They were subsequently used for hematoxylin and eosin (HE) staining according to the manufacturer’s instructions (C0105S, Beyotime) for histological analysis. For pharmacological intervention experiments, 4-month-old WT male mice were treated with intraperitoneal injections of rapamycin (HY-10219, MCE) at a dosage of 5 mg/kg (injection volume: 10 mL/kg) for 5 consecutive days. In contrast, 4-month-old syna CKO male mice were treated with a single intraperitoneal injection of IGF-1 (P5502, Beyotime) at a dosage of 1.5 mg/kg (injection volume: 10 mL/kg) for 30 minutes. Then, testis tissues were fixed, embedded, sectioned, and performed IF and TUNEL staining (GK10041, Glpbio).

#### Mouse sperm collection

Tissues from the caudal epididymal of WT and syna CKO male mice were isolated and placed in 1 ml PBS. The tissues were cut with surgical scissors and incubated at 37°C for 20 minutes to allow the sperm to flow out naturally. Sperm were coated on slides for sperm motility analyses, and sections were subjected to morphological analyses and immunofluorescence staining after fixing in 4% paraformaldehyde.

#### Immunofluorescence staining

Briefly, for sperm staining, sperm sections were incubated with the primary antibody overnight at 4°C. Next, incubated with the secondary antibodies for 60 minutes and with PNA-FITC (L7381, Sigma) for 60 minutes at room temperature. Sperm nuclei were counterstained with DAPI (P0131, Beyotime). Sperm mitochondria were counterstained with Mito-Tracker Red CMXRos (1:1000 dilution, C1049B, Beyotime). Finally, imaged via the orthogonal fluorescence microscope (Nikon, Japan).

For tissue staining, the antigen was repaired with 1× Sodium Citrate Antigen Retrieval Buffer (with a pH of 6.0). The remaining steps are the same as those for sperm immunofluorescence staining. All detailed information on antibodies is shown in Table S2.

#### Enzyme-linked immunosorbent assay (ELISA)

We performed ELISA to detect the concentrations of testosterone (T) (MM-0569M2), GnRH (MM-0506M2), LH (MM-44039M2), PGE2 (MM-0062M2), and PRL (MM-0553M2), further investigating the developmental and reproductive effects of syna CKO on 4-month-old male mice. All procedures were carried out according to the MEIMIAN company’s protocol. The concentration value of each sample was calculated according to the curve equation by plotting the linear regression curve of the standard.

#### Flow cytometry analysis

Single-cell suspensions were obtained from mouse peripheral blood and testis tissues, and flow cytometry was used to analyze individual subsets. The white blood cell marker CD45 (147703, Biolegend), the T cell markers: CD3 (100203, Biolegend), CD8 (100713, Biolegend), and CD4 (100433, Biolegend), the B cell marker CD19 (115512, Biolegend), and the NK cell marker CD16 (158003, Biolegend), testis Leydig cells marker CD90 (E-AB-F1283D, Elabscience), and Fluo 3-AM (F8840, Solarbio) were analyzed. The experiment was performed by Beckman CytoFlex and analyzed by CytExpert2.5 software.

#### Transmission electron microscopy (TEM) analysis and scanning electron microscopy (SEM) analysis

For the TEM assay, sperm were fixed with 2.5% glutaraldehyde and 1% osmium tetroxide, and then dehydrated with gradient ethanol. Infiltrate the samples overnight with a mixture of acetone and epoxy resin (Epon 812), then infiltrate with pure epoxy resin overnight. Polymerize at 60°C for 48 hours. Cut ultrathin sections of 60-80 nm using an ultramicrotome. Stain the sections with uranyl acetate and lead citrate for 15 minutes each. Imaging under a transmission electron microscope (Hitachi S-4800). For the SEM assay, sperm were fixed with 3-4% glutaraldehyde, dehydrated with gradient ethanol, and dried naturally. Gold was sprayed on the samples with a HITCH E-1010 ion sputterer to increase the conductivity. Imaging was done under the scanning electron microscopes (Hitachi JEM-2100 and Thermo scientific quattro ESEM).^58^

#### RNA sequencing (RNA-Seq) analysis and bioinformatics

Total RNA was extracted from the entire testes of 4-month-old WT and syna CKO male mice using trizol and then sequenced. Sequencing was performed using the Illumina Hiseq™ platform. The quality was assessed by FastQC. Gene co-expression analysis was performed using WGCNA, and the DESeq2 software acquired the differentially expressed genes (DEGs) analysis. The topGO software was used for GO enrichment analysis. The enrichment analyses of the KEGG pathway and KOG classification were performed using the clusterProfiler. The sequencing and data analyses were all performed by Sangon biotech (Shanghai, China).

#### Oil red O staining

Oil red staining of frozen testis sections from 4-month-old syna CKO and WT male mice was performed to observe the effect of syna CKO on lipid metabolism. According to the manufacturer’s instructions, the oil red O staining kit (C0157S, Beyotime) was used.

#### Cell culture and calcium rescue experiment

Primary MEFs were isolated from E14 Syna^ff^ mouse embryos. MEFs were infected with Lenti-EF1α-Cre-P2A-EGFP-Puro (C4102, Beyotime) to generate syna-deficient cells. For the calcium rescue assay, syna-deficient MEFs were treated with the calcium ionophore A23187 (GC11200, Glpbio) (1 μM for 30 min) to induce calcium influx. Cells were then lysed in M-PER mammalian protein extraction reagent (QJ222436, Thermo scientific) supplemented with protease and phosphatase inhibitors. Total protein was extracted, and the expression levels of p-AKT and pmTOR were analyzed by WB.

### Quantification and statistical analysis

Statistical analyses were performed using GraphPad Prism software (version 10.3.1, GraphPad Software, Boston, MA, USA). ImageJ software was utilized for the quantification of grey values in Western blot bands. All statistical details, including the exact value of n and specific tests used, are indicated in the figure legends. Data are presented as the mean ± standard deviation (SD).

Comparisons between two groups (e.g., WT vs. Syna CKO) were analyzed using an unpaired, two-tailed Student’s t-test. Before analysis, the assumption of homogeneity of variance was verified using the F-test (P > 0.05), confirming that the data met the assumptions for the parametric approach. Significance was defined as P < 0.05.
