# Supplementary material for: Syncytin-a deficiency compromises murine sperm function by suppressing PRL/PGE2 and PI3K/AKT/mTOR pathway
Source: iScience. 2026 Apr 12;29(5):115694. doi: 10.1016/j.isci.2026.115694 (PMC13141077; doi:10.1016/j.isci.2026.115694)
Supplement: Document S1. Figures S1–S8 and Tables S1 and S2 [file mmc1.pdf]

iScience, Volume *29*

Supplemental information

Syncytin-a deficiency compromises murine sperm function by suppressing PRL/PGE2 and PI3K/AKT/mTOR pathway

Qianqian Wang, Zhenwei Wang, Zhenpeng Li, Dongle Liu, Xiaotong Yan, Yan Zhang, Ning Chi, Qiang Bian, Zhankui Zhao, and Honglian Yu

Inventory of Supplemental Information

1 Figure S1-S8

2 Table S1 and S2

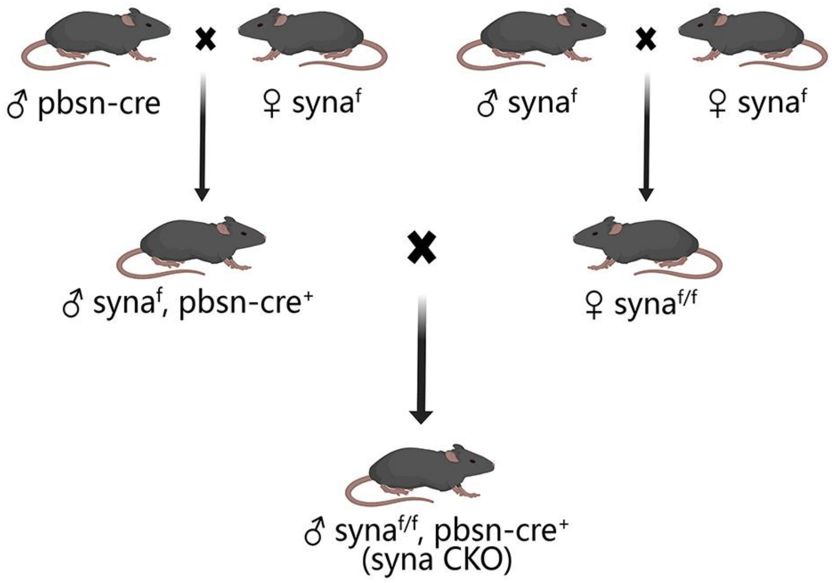

Figure S1. The breeding program of syna CKO male mice.

The syna CKO mice were eventually obtained by breeding the pbsn-cre and synaf mice.

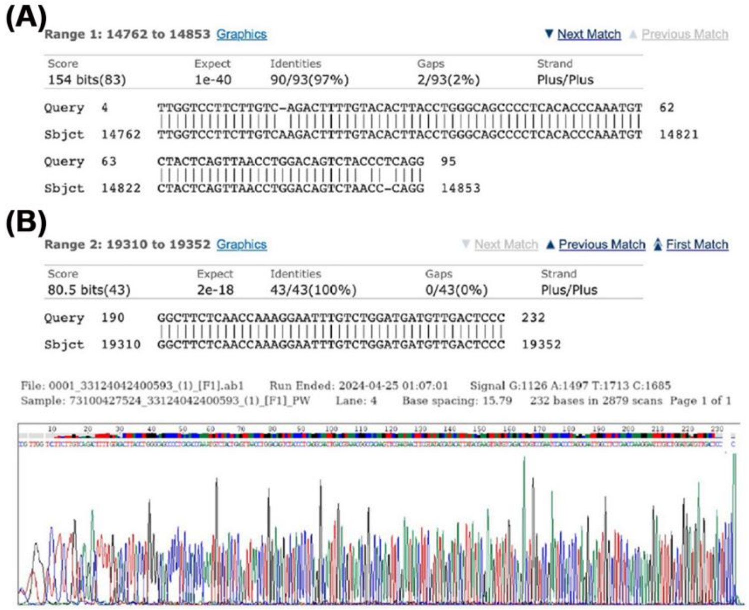

Figure S2. The analysis of gene sequencing.

(A) NCBI Blast result between DNA sequencing sequence and syna gene sequence.

(B) DNA sequencing results in Chromas software.

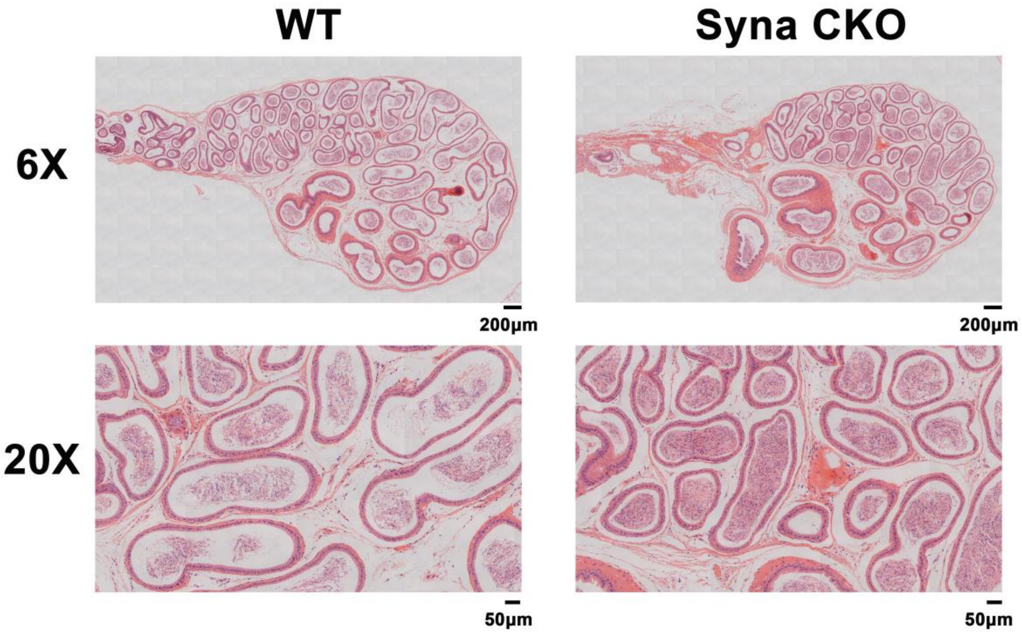

Figure S3. HE staining of the cauda epididymis from WT and syna CKO male mice. Scale bars: 50μm, 200μm.

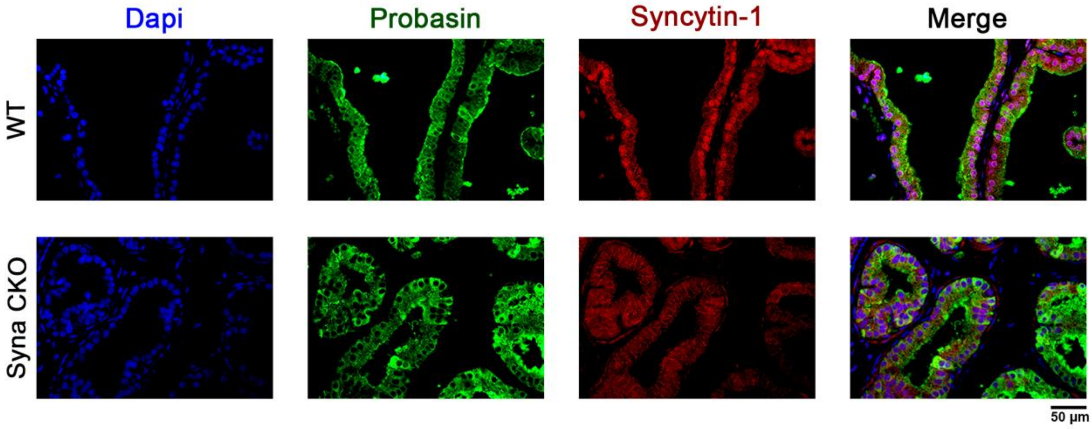

Figure S4. Immunofluorescence staining of the prostate from WT male mice and syna CKO male mice.

Prostate sections were incubated with primary antibodies probasin (green) and syncytin-1 (red), and Dapi (blue). The merged image shows that the expression of syncytin-1 decreased in syna CKO male mice than in WT male mice, and the prostate epithelial secretory cells were loosely arranged in syna CKO mice. Scale bars: 50 μm.

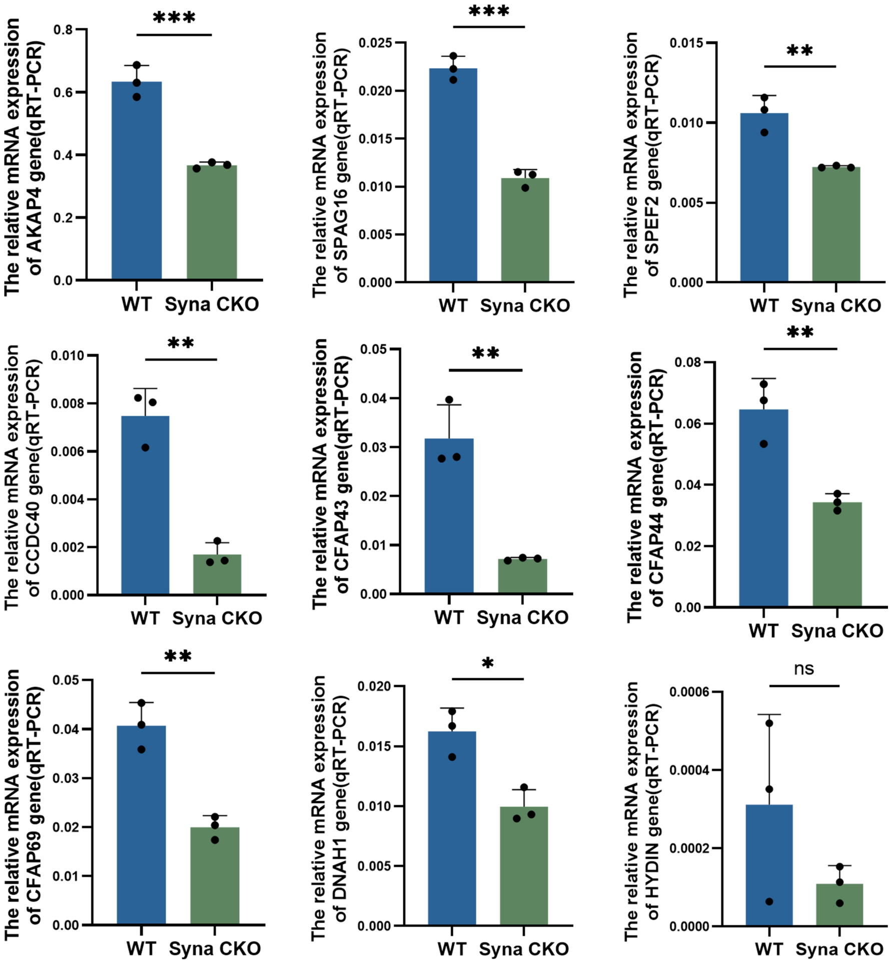

Figure S5. The relative mRNA expression of different genes.

These figures revealed the relative mRNA expression of AKAP4, SPAG16, CCDC40, CFAP43, CFAP44, CFAP69, SPEF2, DNAH1 and HYDIN genes. GAPDH was used as an internal control. Data are presented as mean ± SD of n = 3 biologically independent experiments.

Mean ± standard error is displayed in dot plots and bar charts. ***P<0.001, **P<0.01, *P<0.05.

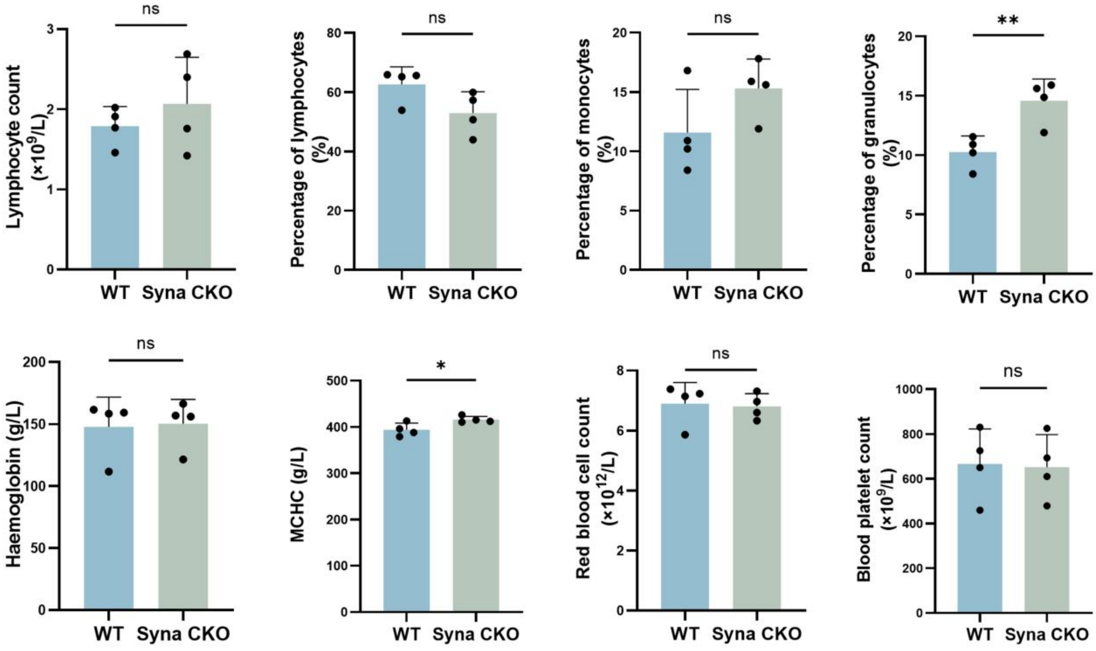

Figure S6. The results of the routine blood test.

The counts of lymphocyte, red blood cells, and blood platelet. The percentage of lymphocytes, monocytes, and granulocytes. And the concentration of haemoglobin and mean corpuscular hemoglobin concentration (MCHC). Data are presented as mean ± SD of n = 4 biologically independent experiments. Mean ± standard error is displayed in dot plots and bar charts. **P<0.01, *P<0.05.

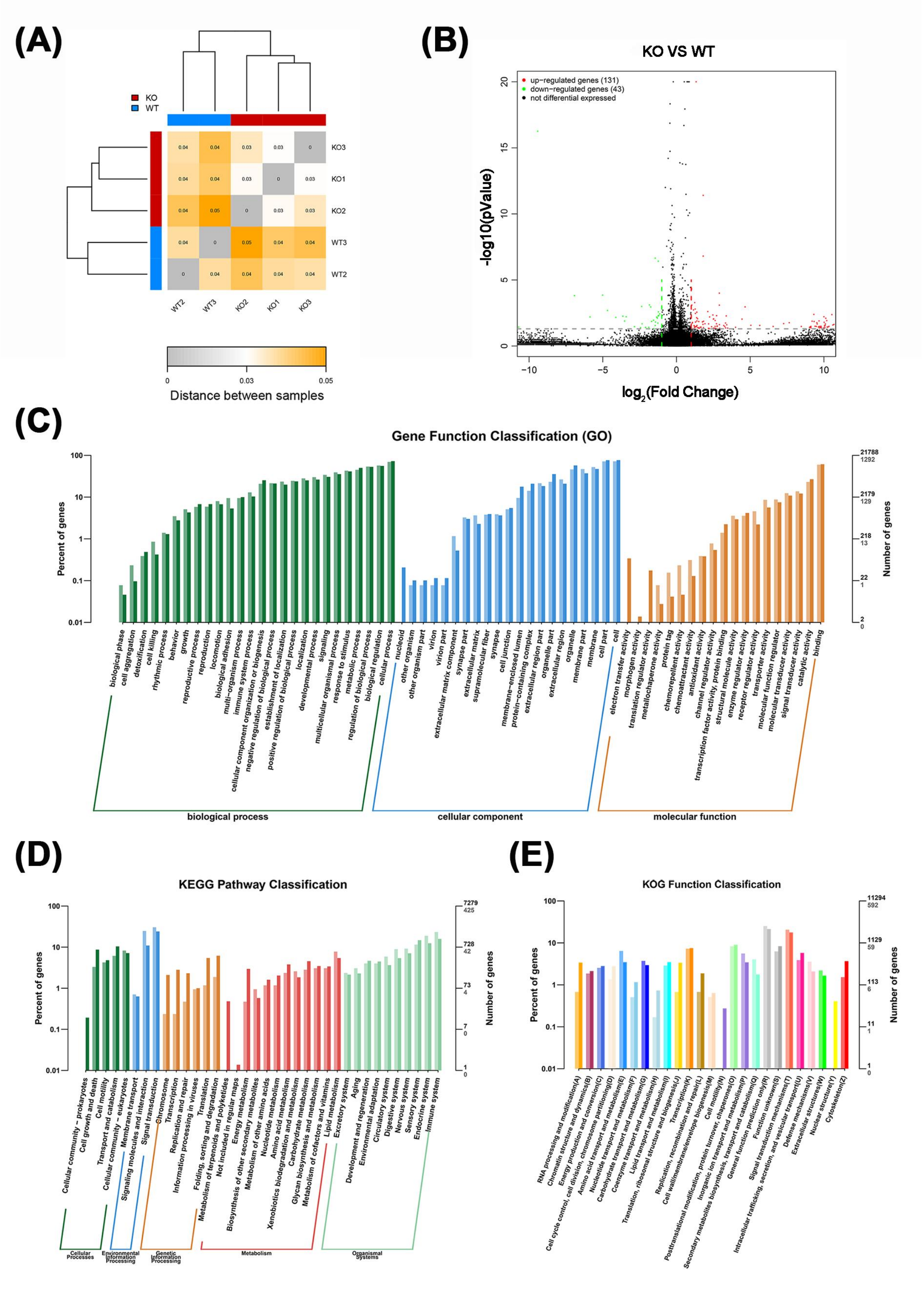

Figure S7. Comparative transcriptomic analysis between knockout (KO) and wild-type (WT) samples.

(A) Heatmap on the distance between WT and syna CKO male mice.

(B) Volcano map on differential genes between WT and syna CKO male mice.

(C) Gene Ontology (GO) classification is divided into three categories: biological process (green bars), cellular component (blue bars), and molecular function (orange bars).

(D) KEGG (Kyoto Encyclopedia of Genes and Genomes) pathway classification, with the genes grouped into different pathway categories such as cellular processes (dark green bars), environmental information processing (blue bars), genetic information processing (orange bars), metabolism (red bars) and organismal systems (light green bars).

(E) KOG (euKaryotic Ortholog Groups) function classification displays genes in various functional categories such as signal transduction, cell cycle control, and metabolism. Values on the bar graph and vertical axis are light for differential genes and dark for all genes. The right vertical axis is the number of genes within the category, and the left vertical axis is the percentage of genes annotated to the function (differential genes/all genes).

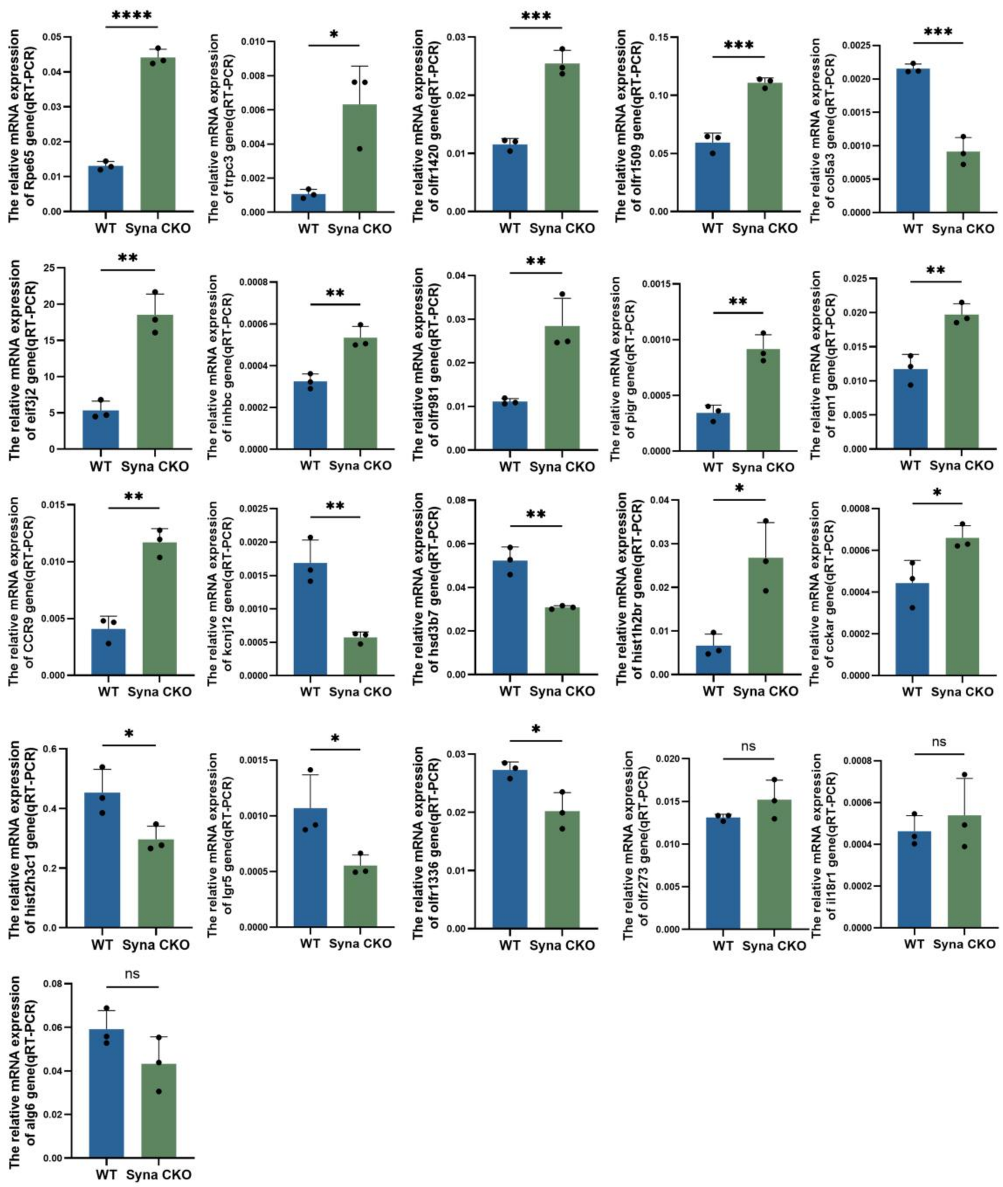

Figure S8. The relative mRNA expression of different genes by qRT-PCR.

The relative mRNA expression of rpe64, trpc3, olfr1420, olfr1509, col5a3, eif3j2, inhbc, olfr981, pigr, ren1, ccr9, kcnj12, hsd3b7, hist1h2br, cckar, hist2h3c1, lgr5, olfr1336, olfr273, il18r1, and alg6 genes. Data are presented as mean ± SD of n = 4 biologically independent experiments.

Mean ± standard error is displayed in dot plots and bar charts. ****P<0.0001, ***P<0.001, **P<0.01, *P<0.05.

2 Table S1 and S2

Table S1 Primers for genotyping and PCR

Gene	Direction		Sequence (5'-3')	Products (bp)
Syna	Loxp-1	F1<br>R1	GGAGCAAAGGCTCTTAGCATCT<br>CACAAACAGACATGCAAGTCACTCA	WT=306; flox/flox=376
	Loxp-2	F2<br>R2	CTCCCTTCCCAAGGTTTATCTCAG<br>GGGAGTCAACATCATCCAGACAAA	WT=149; flox/flox=216
	Loxp	F1<br>R2	GGAGCAAAGGCTCTTAGCATCT<br>GGGAGTCAACATCATCCAGACAAA	265
Cre	F<br>R		CATATTGGCAGAACGAAAACGC<br>CCTGTTTCACTATCCAGGTTACGG	413
Synb	F<br>R		TGCTTGTCTACCCAAACCCAG<br>GGCCTGACTGAGTAGGTAAGATG	101
α tubulin	F<br>R		TGTGGATTCTGTGGAAGGCG<br>AAGCACACATTGCCACATACAA	150
Syna	F<br>R		ATGGTTCGTCCTTGGGTTTTC<br>GTGTTGAGTGAGGTTTACCAGG	93
GAPDH	F<br>R		TGGCCTTCCGTGTTCCTAC<br>GAGTTGCTGTTGAAGTCGCA	178

Syna	F<br>R	ATGGTTCGTCCTTGGGTTTTC<br>GTGTTGAGTGAGGTTTACCAGG	93
Synb	F<br>R	TGCTTGTCTACCCAAACCCAG<br>GGCCTGACTGAGTAGGTAAGATG	101
AKAP3	F<br>R	ATGGCGGATAGGGTTGACTG<br>CACTCGGACAGGATCTGTTGA	123
AKAP4	F<br>R	GTCAGAAGGCGAGTTAAATCTGG<br>ATCCCTCCGTCTTAGACTGGT	95
DNAH1	F<br>R	CGCCTGTCTTCTGGATCAGT<br>ATTTCTGATGACGCCTCGGG	142
ENO4	F<br>R	AGGACGGAGGGATACGCTTTT<br>AGGGTTAAGTAGAGAGGATCGC	150
HYDIN	F<br>R	AAAATCCCAAACGTGCTCTTCT<br>GGGTGGTAAGACTTTGCTCTG	189
SPEF2	F<br>R	GACCGTGAGTCCCAAGTCATT<br>GCAAGCGGGAGAAATTGTTAAG	149
SPAG6	F<br>R	GGTGGACGCAGGAGCTATTG<br>CTCTGCTTCAACAACCATCTCT	139
SPAG16	F<br>R	CCAGATCCAAACGTGACTTCA<br>CCAGACAACCAATCAGTGTGG	227
CFAP69	F<br>R	AACAGCAGTTGGATGTCCGC<br>AGGCTTGAAAATACCCTGCATC	293
CFAP43	F<br>R	GCTTCTGGAATTGATGGTGTGA<br>GACAGCCTGCACTTTCCCA	228
CFAP44	F<br>R	AAGAGTCGTATCTGGGCGATG<br>CACCTTCTTTACGTCGGCTTC	146

CCDC39	F<br>R	AATCGGCTCACAAAGACAGTG<br>GGAAATCCTGTGCAGCTTTATCT	195
CCDC40	F<br>R	ATCAACCGACACATTCCAGCA<br>TGTCCGAGGGAGTCAGACTTC	212

Table S2 The antibodies used in the manuscript

Antibody	Type	Host	Company	Cat No.	Dilution
Syncytin-1	Polyclonal	Rabbit	Bioss	bs-2962R	1:1500 (WB)
ERVFRD1	Polyclonal	Rabbit	Bioss	bs-15466R	1:1500 (WB)<br>1:300 (IF)
ENO4	Polyclonal	Rabbit	Elabscience	E-AB-17910	1:1500 (WB)
CCDC39	Polyclonal	Rabbit	Affinity	DF13893	1:500 (WB)
SPAG6	Polyclonal	Rabbit	Proteintech	12462-1-AP	1:1000 (WB)
AKAP3	Polyclonal	Rabbit	Proteintech	13907-1-AP	1:5000 (WB)
GAPDH	Polyclonal	Rabbit	Affinity	AF7021	1:3000 (WB)
β-actin	Polyclonal	Rabbit	Affinity	AF7018	1:3000 (WB)
Phospho-AKT1	Polyclonal	Rabbit	Abcepta	AP3434a	1:1000 (WB)
Akt	Monoclonal	Rabbit	Cell Signalling Technology	4691S	1:1000 (WB)
Phospho-STAT3	Monoclonal	Rabbit	Abcam	ab76315	1:10000 (WB)
STAT3	Monoclonal	Mouse	Abcam	ab119352	1:5000 (WB)
p44/42 MAPK (Erk1/2)	Monoclonal	Rabbit	Cell Signalling Technology	4695S	1:500 (WB)
Phospho-p44/42 MAPK (pErk1/2)	Monoclonal	Rabbit	Cell Signalling Technology	4370S	1:1000 (WB)
Phospho-mTOR	Monoclonal	Rabbit	Cell Signalling Technology	5536P	1:500 (WB)
mTOR	Monoclonal	Rabbit	Cell Signalling Technology	2983P	1:500 (WB)
Phospho-4E-BP1	Monoclonal	Rabbit	Cell Signalling Technology	2855P	1:500 (WB)
Anti-Bcl-2	Polyclonal	Rabbit	Abcam	ab196495	1:1000 (WB)

Cleaved-Caspase 3	Polyclonal	Rabbit	Affinity	AF7022	1:1000 (WB)
Cyclin D1	Monoclonal	Rabbit	Boster	BM4272	1:1000 (WB)
Phospho-JNK1/2/3	Polyclonal	Rabbit	Affinity	AF3318	1:1000 (WB)
JNK1/2/3	Polyclonal	Rabbit	Affinity	AF6319	1:1000 (WB)
PI3 Kinase p110α	Monoclonal	Rabbit	Omnimabs	OM644122	1:1000 (WB)
Syncytin-1	Polyclonal	Rabbit	Biorbyt	orb100573	1:300 (IF)
β-Tubulin	Monoclonal	Mouse	Affiniity	T0023	1:500 (IF)
